# KLF4 activates NFκB signaling and esophageal epithelial inflammation via the Rho-related GTP-binding protein RHOF

**DOI:** 10.1371/journal.pone.0215746

**Published:** 2019-04-18

**Authors:** Khvaramze Shaverdashvili, Jennie Padlo, Daniel Weinblatt, Yang Jia, Wenpeng Jiang, Divya Rao, Dorottya Laczkó, Kelly A. Whelan, John P. Lynch, Amanda B. Muir, Jonathan P. Katz

**Affiliations:** 1 Division of Gastroenterology, University of Pennsylvania Perelman School of Medicine, Philadelphia, United States of America; 2 Division of Gastroenterology, Hepatology and Nutrition, The Children’s Hospital of Philadelphia, Philadelphia, United States of America; University of Central Florida College of Medicine, UNITED STATES

## Abstract

Understanding the regulatory mechanisms within esophageal epithelia is essential to gain insight into the pathogenesis of esophageal diseases, which are among the leading causes of morbidity and mortality throughout the world. The zinc-finger transcription factor *Krüppel*-like factor (KLF4) is implicated in a large number of cellular processes, such as proliferation, differentiation, and inflammation in esophageal epithelia. In murine esophageal epithelia, *Klf4* overexpression causes chronic inflammation which is mediated by activation of NFκB signaling downstream of KLF4, and this esophageal inflammation produces epithelial hyperplasia and subsequent esophageal squamous cell cancer. Yet, while NFκB activation clearly promotes esophageal inflammation, the mechanisms by which NFκB signaling is activated in esophageal diseases are not well understood. Here, we demonstrate that the Rho-related GTP-binding protein RHOF is activated by KLF4 in esophageal keratinocytes, leading to the induction of NFκB signaling. Moreover, RHOF is required for NFκB activation by KLF4 in esophageal keratinocytes and is also important for esophageal keratinocyte proliferation and migration. Finally, we find that RHOF is upregulated in eosinophilic esophagitis, an important esophageal inflammatory disease in humans. Thus, RHOF activation of NFκB in esophageal keratinocytes provides a potentially important and clinically-relevant mechanism for esophageal inflammation and inflammation-mediated esophageal squamous cell cancer.

## Introduction

Esophageal diseases are among the leading causes of morbidity and mortality in the U.S. and the world [1]. For example, esophageal cancers, of which approximately 90% are esophageal squamous cell cancer (ESCC) [2], are the 8^th^ most common cause of cancer and the 6^th^ leading cause of cancer-related deaths worldwide [3, 4]. Many diseases of the squamous esophagus, including ESCC and eosinophilic esophagitis (EoE), occur in the setting of chronic inflammation, and a number of these conditions have been effectively modeled in the mouse, leading to new insights into molecular pathogenesis of these diseases [5–10]. In particular, the NFκB signaling pathway has emerged as a critical activator of epithelial inflammation and inflammation-mediated carcinogenesis [11, 12], and in the esophagus, activated NFκB signaling is implicated in the development of ESCC and EoE, among other disorders [7, 13–20]. Moreover, constitutive NFκB activation in murine esophagus promotes inflammation and angiogenesis *in vivo* [6]. However, to date, the molecular mechanisms governing NFκB pathway activation in esophageal epithelia are not well understood.

In murine esophagus, upregulation of the DNA-binding transcription factor *Krüppel*-like factor 4 (KLF4) within squamous epithelial cells activates NFκB signaling, leading to chronic inflammation and inflammation-mediated ESCC [7]. KLF4 has other important cell-autonomous functions, including in proliferation and differentiation, and pro-inflammatory effects of KLF4 in esophageal epithelia are consistent with those seen in other cell-types and tissues [21–31]. For example, KLF4 promotes macrophage polarization and signaling, monocyte differentiation, cytokine expression in dendritic cells, vascular inflammation, inflammatory responses in microglia, and intestinal inflammation via NFκB signaling. Classically, the NFκB pathway is stimulated by proinflammatory cytokines or other receptor ligands, leading to the activation of IκB kinase (IKK) which then phosphorylates IκB, leading to IκB degradation and subsequent nuclear translocation of canonical NFκB members [11, 32]. Yet the mechanisms by which the transcription factor KLF4 activates NFκB signaling are not known.

Previously, we conducted a microarray analysis for genes differentially regulated in the presence and absence of *Klf4* in murine esophagus and identified the Rho GTPase *RhoF* as a potential KLF4 target [33]. Rho GTPases can activate NFκB signaling, including in the esophagus and the skin, which like the esophagus is lined by a squamous epithelium, making Rho GTPases intriguing candidates as downstream mediators of KLF4 on NFκB and inflammation [34–38]. RHOF (also known as RIF) is a Rho family member that has been implicated in membrane trafficking, cell migration, and cytoskeletal dynamics [39–43]. Like other Rho GTPases, RHOF cycles between an active GTP-bound state and an inactive GDP-bound state, a process mediated by the interplay between guanine nucleotide exchange factors (GEFs) and the opposing GTPase activating proteins (GAPs) [39, 44, 45]. Interestingly, constitutive *RhoF* knockout mice have no overt phenotype, suggesting that RHOF may be dispensable for cellular function *in vivo* under normal conditions [46]. A role for RHOF in inflammation has not previously been reported.

Here we show that RHOF is upregulated by KLF4 and that RHOF promotes inflammation and is required for induction of NFκB signaling by KLF4 in esophageal keratinocytes. In addition, we find that RHOF is increased in a human esophageal inflammatory disease. Thus, we demonstrate that RHOF as an important mediator of esophageal inflammation.

## Materials and methods

### Immunohistochemistry

Murine primary esophageal keratinocytes isolated from *ED-L2/Klf4* mice or *ED-L2/Cre;Klf4*^*loxp/loxp*^ mice and age-matched controls, both male and female, were used for experiments [7, 33]. Mice were housed in the barrier facility at the University of Pennsylvania and given *ad libitum* access to food and water. All animal studies were approved by the Institutional Animal Care and Use Committee at the University of Pennsylvania (IACUC) under protocol #803502. Human subjects were enrolled at the time of diagnostic esophagogastroduodenoscopy at the Hospital of the University of Pennsylvania (IRB #813363). Inclusion criteria for initial recruitment included no other esophageal or chronic inflammatory disease of the gastrointestinal tract. EoE subjects were diagnosed based on 2011 clinical guidelines [47]. Non-EoE subjects are comprised of patients who reported symptoms warranting upper endoscopy, did not carry a previous diagnosis of EoE and demonstrated no histopathologic abnormalities. For immunohistochemistry, human and mouse tissues or organotypic cultures were processed using standard protocols described elsewhere [33, 48]. Paraffin embedded slides were stained using rabbit anti-KLF4 [49] at 1:2,500 dilution and rabbit anti-RHOF antibodies (LS Bio,Lot# 66566, SL C353833) at 1:300 dilution.

### Western blots

Western blots were performed as described previously [33, 50]. Briefly, cells were lysed with Triton Lysis buffer, and protease and phosphatase inhibitors (Sigma-Aldrich) were added. From each sample, 15 μg of protein was separated on a NuPAGE 4–12% acrylamide gel (Invitrogen). The following antibodies were used for Western blotting: rabbit anti-KLF4 antibody [49] at 1:10,000 dilution; rabbit anti-RHOF antibody (LSBio LS-C353833) at 1:500 dilution; rabbit anti-phospho-p65 (Cell Signaling, S536) at 1:1,000 dilution; rabbit anti-p65 (Cell Signaling, C22B4) at 1:1,000 dilution; mouse anti-IKK2 (Cell Signaling) at 1:1,000 dilution; mouse anti-β-actin at 1:10,000 dilution; rabbit anti-GAPDH (Cell Signaling) at 1:10,000 dilution; and mouse anti-α-tubulin at 1:15,000 dilution as described previously [50].

### RNA analyses and real-time PCR

Total RNA was isolated with the RNeasy Micro Kit (Qiagen) following manufacturer’s protocol, and cDNA was synthesized with the Maxima First Strand cDNA Synthesis Kit for RT-qPCR, with dsDNase (ThermoFisher). Quantitative real-time polymerase chain reaction (qPCR) was performed using SYBR Green Master Mix (ThermoFisher) as described [50]. Relative mRNA expression levels were normalized by GAPDH. The following primer were used to amplify specific target genes: *RHOF* (human F 5’-AGCAAGGAGGTGACCCTGAAA-3’, R 5’-CCGCAGCCGGTCATAGTC-3’; mouse F 5’-ACTGCTCCTTGTCCTTCCTCA-3’, R 5’-CGACAACGTCCTCATCAAAGTG-3’);

KLF4 (human F 5’-GCGGCAAAACCTACACAAAG-3’, R 5’-CCCCGTGTGTTTACGGTAGT-3’; mouse F 5’-GTGCCCCGACTAACCGTTG-3’ R 5’-GTCGTTGAACTCCTCGGTTCT-3’);

IKK1 (human F 5’-CTCCGAGACTTTCGAGGAAATAC-3’, R 5’-GCCATTGTAGTTGGTAGCCTTCA-3’; mouse F 5’-GTCTCGGAATTGAGCGTGAAA-3’, R 5’-TCCCTGTCTCTGACAGAAGCTCCTGA-3’); IKK2 (mouse F 5’-TCTAAATGGCCTTTTCCTGCTAAT-3’, R 5’-TGACTCTCCCAAAGTTAGATGCA-3’);

IKK3 (mouse F 5’-CTGGAAGATCTGAGGCAACA-3’, R 5’-CCAGGGCCTCCTCAGCTTGC-3’); GAPDH (human F 5’-GAAGGTGAAGGTCGGAGTCA-3’ R 5’-AATGAAGGGGTCATTGATGG-3’; mouse F 5’-CGGCCGCATCTTCTTGTG-3’ R 5’-ACCGACCTTCACCATTTTGTCT-3’.

### Cell culture and treatment

Murine primary esophageal keratinocytes were grown as described [7, 33, 50]. Established primary human esophageal keratinocytes (EPC2-hTERT cells) [51] were grown in keratinocyte serum-free medium (K-SFM, Invitrogen) supplemented with bovine pituitary extract and epidermal growth factor (Invitrogen) [48]. Cells were infected and transfected as described [48]. For cytokine studies, cells were stimulated with 10 ng/ml IL-4, IL-13, or TGFβ (R&D Systems) for 24 hours.

### shRNAs and expression plasmids and transfection

Full-length constitutively active *RhoF* (myc Rif-QL) [43] was a gift from Harry Mellor (Addgene plasmid #38768) and was overexpressed in mouse epithelial cells using lentiviral vectors psPAX2 (gift from Didier Trono, Addgene plasmid #12260) and pMD2.G (gift from Didier Trono, Addgene plasmid #12259. Two distinct shRNAs against *RhoF* (Sigma, TRCN0000077678 and TRCN0000447186) were used for knockdown experiments. FuGENE 6 (Promega) was used for lentiviral generation.

### RHOF activation assay

RHOF activation assays were performed using protocols described elsewhere [42, 52]. The GST-MDia1 plasmid (gift of Harry Mellor) was used to generate GST-MDia1 protein, which was used as a probe to specifically isolate the active form of RHOF. Esophageal keratinocytes from *ED-L2/Klf4* mice were seeded on 100 mm culture dishes and lysed. RHOF protein was pulled down using GST beads, and the beads were washed three times with washing buffer. Activated RHOF bound to the beads or total RHOF in cell lysates was detected by Western blot using rabbit anti-RHOF antibody (LSBio) at 1:1,000 dilution.

### Organotypic culture

Organotypic culture was performed as previously described [48, 53]. Human esophageal keratinocytes were seeded on a collagen fibroblast layer with and without human peripheral blood mononuclear cells (PBMC), which were stimulated by inflammatory cytokines (IL-2, IL-7, and IL-15; Human Immunology Core, University of Pennsylvania) as described [54]. To model a T_H_1 inflammatory environment, the pro-inflammatory cytokines IL-7 (10 ng/mL; Cell Signaling) and IL-15 (20 ng/mL; Prospec-Tany Technogene) were included in cell culture media, and IL-2 (10 U/mL, BD Biosciences) was added to support PBMC viability. Organotypic cultures were processed and stained according to standard protocols [48].

### Time-lapse video microscopy

Single cells were plated as described previously and cultured on 8-well chamber slides [55, 56]. Cells were kept at 37°C and 5% CO_2_ for the duration of the 24 hour time-lapse recording. Serial phase-contrast images were captured at 10 minute intervals and built into a movie using MetaMorph software (Molecular Devices). Approximately 10 cells per field for a total of three fields per sample were highlighted and their movements followed over the 24 hour period. Distance from the origin was computed using MetaMorph as previously described [55, 57]

### Statistical analysis

Statistical significance between groups of data was calculated by Student's *t* test, using Prism 4 (GraphPad), and differences were considered significant for p <0.05.

## Results

### KLF4 activates NFkB signaling in esophageal keratinocytes

**Fig 1:**
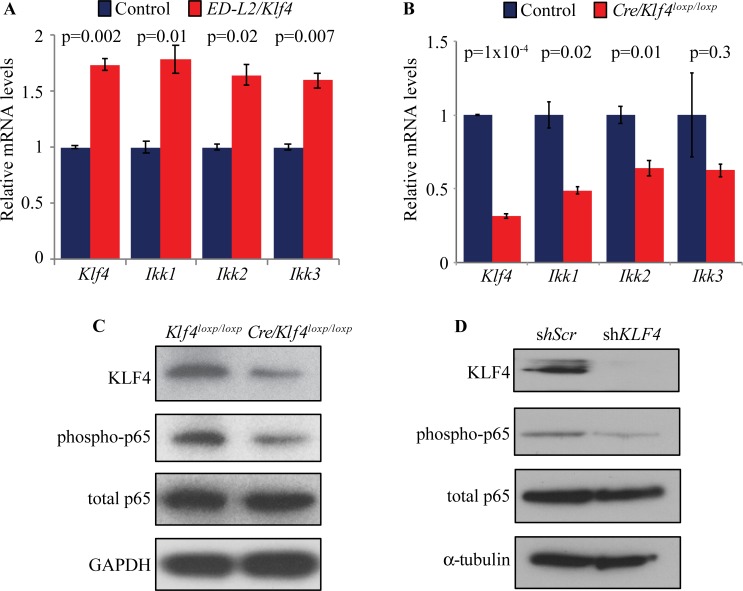
KLF4 activates NFκB signaling in esophageal epithelial cells. (**A**) By qPCR, *Ikk1*, *Ikk2*, and *Ikk3* expression was increased in primary esophageal keratinocytes from mice overexpressing *Klf4* (*ED-L2/Klf4* mice) compared to keratinocytes from control mice. (**B**) By qPCR, expression of *Ikk1*, *Ikk2*, and *Ikk3* was reduced in primary esophageal keratinocytes from mice with loss of *Klf4* (*ED-L2/Cre;Klf4*^*loxp/loxp*^ mice) compared to control keratinocytes from *Klf4*^*loxp/loxp*^ mice without Cre. (**C**) Compared to control keratinocytes from *Klf4*^*loxp/loxp*^ mice without Cre, primary esophageal keratinocytes from *ED-L2/Cre;Klf4*^*loxp/loxp*^ mice had less phosphorylated p65 on Western blots but no change in total p65. (**D**). Compared to a scrambled shRNA control (shSCR), shRNA directed against *KLF4* reduced p65 phosphorylation in primary human esophageal keratinocytes, as seen on Western blot.

Initially, we sought to validate that KLF4 activates NFκB in esophageal epithelial cells. Consistent with our prior work [7], we found that primary murine esophageal keratinocytes from *ED-L2/Klf4* mice, which overexpress *Klf4* in esophageal epithelial cells, had increased expression of the kinases *Ikk1*, *Ikk2*, and *Ikk3*, which activate NFκB signaling, compared to control cells (Fig 1A), and in primary murine esophageal keratinocytes from mice with genetic ablation of *Klf4* (*ED-L2/Cre;Klf4*^*loxp/loxp*^ mice), *Ikk1*, *Ikk2*, and *Ikk3* expression was decreased (Fig 1B). Moreover, NFκB signaling was also decreased as demonstrated by reduction in phosphorylation of the p65 subunit of NFκB, in primary murine esophageal keratinocytes from *ED-L2/Cre;Klf4*^*loxp/loxp*^ mice (Fig 1C). *Klf4* overexpression in mice activates NFκB [7], and *KLF4* knockdown in primary human esophageal keratinocytes reduced p65 phosphorylation (Fig 1D). Thus, KLF4 upregulates *Ikk1*, *Ikk2*, and *Ikk3* transcription to activate pro-inflammatory NFκB signaling in esophageal epithelial cells, and KLF4 loss decreases NFκB pathway activation. However, the mechanism of this NFκB pathway activation is not known.

### KLF4 increases expression and activity of the small GTPase RHOF

**Fig 2:**
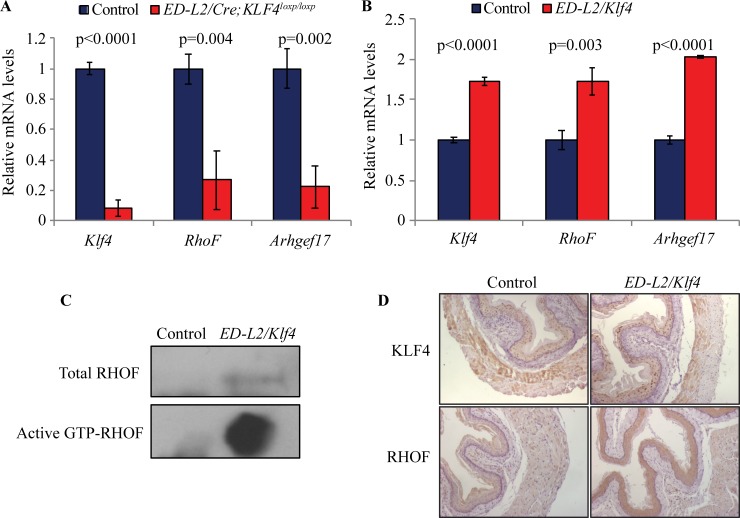
KLF4 increases *RhoF* expression and activity in esophageal epithelial cells. (**A**) By qPCR, *Klf4*, *RhoF*, and the guanine-exchange factor *Arhgef17* were significantly decreased in esophageal epithelial cells from mice with *Klf4* deletion, compared to cells from control mice. (**B**) When *Klf4* was increased in esophageal epithelial cells from mice with *Klf4* overexpression, *RhoF* and *Arhgef17* were increased on qPCR, compared to control cells. (**C**) Esophageal epithelial cells from mice with *Klf4* overexpression also demonstrated an increase in the amount of activated RHOF, indicated on a RHOF activation assay. (**D**) Staining for RHOF (brown) was low in esophageal epithelia of control mice and increased markedly in mice with *Klf4* overexpression. Magnification = 100x.

To define the factors in esophageal epithelial cells that mediate NFκB pathway activation by KLF4, we examined a list of candidate KLF4 targets previously identified by microarray analysis [33], focusing on those with potential functions in NFκB activation. Certain Rho GTPases can activate NFκB signaling [34–38], and both *RhoF* (also known as *Rif*) and *Argef17*, which encodes RhoGEF17, a Rho-specific guanine nucleotide exchange factor (GEF) [58], are differentially expressed by microarray in esophageal epithelia of mice with *Klf4* deletion [33]. RHOF functions in cytoskeletal remodeling but has not previously been implicated in inflammatory signaling [37, 41, 59]. Nonetheless, we postulated that RHOF might mediate NFκB pathway activation by KLF4. By qPCR, we demonstrated that *RhoF* and *Argef17* were downregulated in esophageal epithelial cells from mice with *Klf4* loss (Fig 2A) and increased in esophageal epithelial cells from with *Klf4* overexpression (Fig 2B). Rho factors cycle between an active GTP-bound state and an inactive GDP-bound conformation [44], and to determine whether upregulation of *RhoF* by KLF4 also results in an increase in activated RHOF, we determined the levels of total and GTP-bound RHOF using a GTP pull-down assay [52] in esophageal epithelial cells from control mice and mice with esophageal epithelial *Klf4* overexpression. We found that *Klf4* overexpression results not only in more total RHOF but also in a dramatic increase in activated RHOF (Fig 2C). Using *ED-L2/Klf4* mice, which have epithelial-specific *Klf4* overexpression [7], we demonstrated that KLF4 also regulates RHOF expression *in vivo*, as RHOF is increased specifically within esophageal epithelia of *ED-L2/Klf4* mice (Fig 2D). Thus, KLF4 upregulates RHOF in esophageal epithelia, resulting in increased levels of activated RHOF.

### RHOF is upregulated in inflammation and regulates expression of pro-inflammatory cytokines

**Fig 3:**
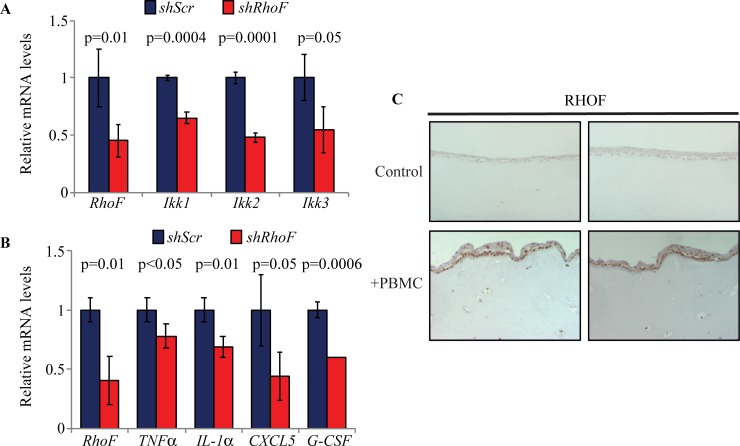
RHOF is upregulated in inflammation and activates pro-inflammatory cytokines. (**A**) By qPCR, expression of the NFκB activators *Ikk1*, *Ikk2*, and *Ikk3* decreased when *RhoF* was knocked down by shRNA in primary esophageal epithelial cells from wild-type mice, compared to similar cells infected with scrambled controls (shScr). (**B**) Compared to cells with shScr, primary mouse esophageal epithelial cells with shRNA against *RhoF* also had reduced expression of pro-inflammatory *TNFα*, *IL-1α*, *CXCL5*, and *G-CSF*. (**C**) When human primary esophageal epithelial cells were grown in organotypic culture with PBMCs that were stimulated with IL-2, IL-7, and IL-15, RHOF levels increased markedly within epithelial cells, compared to cells grown without PBMCs.

To define the function of RHOF in esophageal epithelial cells, we initially examined the effects of *RhoF* knockdown on the actin cytoskeleton and esophageal epithelial cell migration, since RHOF is implicated in cytoskeletal remodeling [41] and Rho GTPases are critical for cell migration [60]. In esophageal epithelial cells, expression of constitutively active RHOF promoted actin remodeling and significantly increased single cell migration (S1 Fig). To define the role of RHOF in esophageal mucosal inflammation, we first knocked down *RhoF* in esophageal epithelial cells using shRNA and examined the consequences on pro-inflammatory genes. *RhoF* knockdown significantly decreased expression of *Ikk1*, *Ikk2*, and *Ikk3* (Fig 3A), consistent with a function for RHOF in mediating KLF4 activation of NFκB signaling. In addition, *RhoF* knockdown reduced expression of the key pro-inflammatory genes *TNFα*, *IL-1α*, *CXCL5*, and *G-CSF* (Fig 3B), each of which is also upregulated in esophageal epithelial cells with *Klf4* overexpression [7]. Finally, RHOF was upregulated in a model of esophageal inflammation (Fig 3C), in which PBMCs stimulated with interleukins are co-cultured with esophageal epithelial cells in organotypic culture [53]. Thus, RHOF is induced in esophageal epithelia during inflammation and activates NFκB signaling to promote inflammation.

### RHOF mediates NFκB pathway activation by KLF4

**Fig 4:**
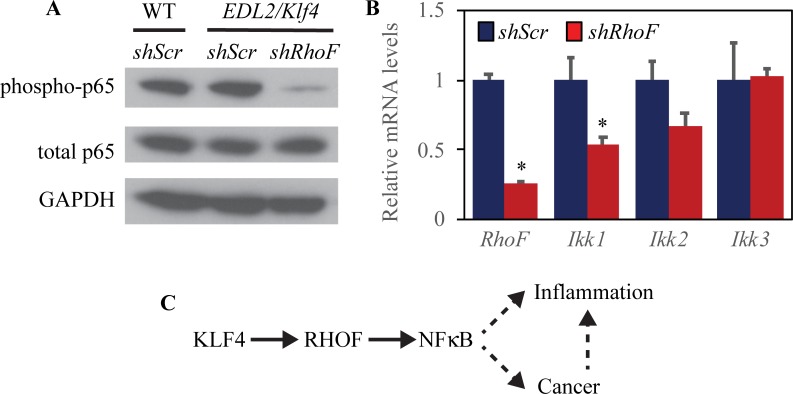
Knockdown of *RhoF* prevents KLF4-mediated activation NFκB. (**A**) By Western blot, *RhoF* knockdown with shRNA blocked p65 phosphorylation in primary esophageal epithelial cells from mice with *Klf4* overexpression. (**B**) By qPCR, *RhoF* knockdown in mouse esophageal epithelial cells with *Klf4* overexpression reduced *Ikk1* and *Ikk2* expression (*p≤0.05). (**C**) KLF4 upregulates RHOF and increases RHOF activation in esophageal epithelia, leading to NFκB activation, inflammation, and cancer.

To delineate whether RHOF was required for KLF4 induction of NFκB signaling in esophageal epithelial cells, we infected primary esophageal epithelial cells from mice with *Klf4* overexpression to express shRNA directed against *RhoF* or a scrambled shRNA control. Compared to control esophageal epithelial cells (with endogenous *Klf4* expression) and *ED-L2/Klf4* cells (that overexpress *Klf4*), *ED-L2/Klf4* cells with *RhoF* knockdown had dramatically decreased NFκB activation (Fig 4A) and decreased expression of *Ikk1* and *Ikk2* (Fig 4B). Based on these findings, we propose a model (Fig 4C) in which KLF4 acts via RHOF to induce NFκB signaling, leading to esophageal epithelial inflammation and esophageal squamous cell cancer [7].

### RHOF is upregulated in human eosinophilic esophagitis

**Fig 5:**
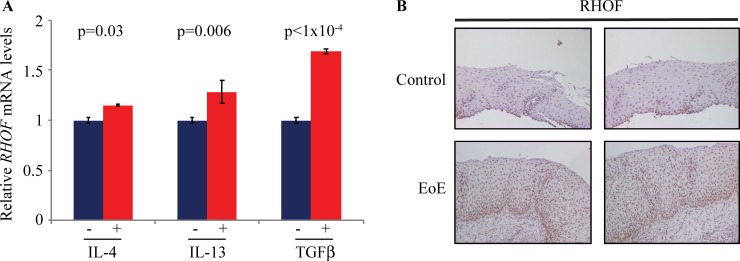
RHOF is upregulated in human EoE. (**A**) Primary human esophageal epithelial cells stimulated with IL-4, IL-13, or TGF-β, cytokines that are physiologically relevant for human EoE, had significant increases in *RHOF* expression by qPCR, compared to unstimulated cells. (**B**) By immunohistochemistry, RHOF levels increased dramatically in EoE compared to normal esophageal epithelia.

To determine whether RHOF might have a role in human esophageal inflammation, we examined the expression of RHOF in an *in vitro* model of the human inflammatory disease eosinophilic esophagitis (EoE) and in human EoE samples. When primary human esophageal epithelial cells were treated with IL-4, IL-13, or TGFβ, cytokines that are upregulated in human EoE [61, 62], *RHOF* increased significantly, including a nearly two-fold increase following TNFβ treatment (Fig 5A). In addition, RHOF was found at much higher levels in esophageal epithelia from humans with EoE, compared to controls (Fig 5B). Thus, elevated RHOF expression is observed in human EoE and *in vitro* EoE models.

## Discussion

In the esophagus, activation of proinflammatory pathways within esophageal squamous epithelial cells can promote inflammation throughout the mucosa, providing a microenvironment favorable for the development of ESCC [5–8]. Previously, we demonstrated that transgenic overexpression of *Klf4* within esophageal keratinocytes activates NFκB signaling, which is associated with the development numerous inflammatory diseases and cancers [11–20], resulting in inflammation-mediated ESCC [7]. We also showed that, consistent with this, activation of NFκB signaling within esophageal keratinocytes by transgenic *Ikkβ* expression promotes inflammation and angiogenesis, features of inflammatory diseases and the tumor microenvironment [5, 63, 64]. The Rho GTPases interact with the NFκB pathway and are involved in the pathogenesis of a number of human cancers and other inflammatory diseases [35]. Here, we link the Rho family member RHOF to KLF4-mediated NFκB activation in esophageal keratinocytes and to the development of inflammation and a human esophageal inflammatory disease, EoE.

Tumor-promoting inflammation is an “enabling characteristic” of cancers, including ESCC [5, 63], and to date, a number of important murine models for inflammation-mediated ESCC have been developed, including mice with *Klf4* overexpression, *p120* catenin knockout, or conditional *Sox2* knockout [7, 8, 65]. Both *Klf4* overexpressing mice and *p120* catenin knockout have robust NFκB activation that is an early event, and in *Sox2* knockout mice, tumor progression correlates with inflammation. Interestingly, *p120* deletion in murine esophagus and epidermis results in inflammation, hyperproliferation, and squamous cell cancer that appear to be mediated by aberrant activation of RHOA upstream of NFκB [36, 38, 66], and Rho GTPases also function in immune cell migration and the tumor microenvironment [67, 68]. Yet similar functions for RHOF in inflammation and tumorigenesis have not previously been reported. Of note, global *RhoF* deletion in mice has no overt phenotype, raising the possibility that other Rho family members may compensate for RHOF function *in vivo* under normal conditions [46].

Interestingly, KLF4 enhances RHOF protein activation, seemingly to a greater extent than can be explained through KLF4 upregulation of *RhoF* expression alone. The mechanism for RHOF activation by KLF4 is not known but may be related to effects of KLF4 on the GEFs and GAPs, the interplay between which regulates the activity of Rho GTPases [37, 39, 44, 45]. In fact, KLF4 does upregulate one of these factors, *Arhgef17*, and increased levels of RhoGEF17 would be expected to increase Rho factor activation. shRNA knockdown of *RhoF* results in a dramatic decrease in phosphorylated p65, some of which is likely related to the effects of KLF4, both endogenous and transgenic, on NFκB signaling, although these data also raise the intriguing possibility that RHOF might promote esophageal inflammation and disease independent of KLF4. Taken together, we conclude that RHOF activates NFκB signaling and esophageal epithelial inflammation, and thus RHOF, and potential activators of RHOF such as RhoGEF17, could emerge as therapeutic targets for inflammatory diseases.

## Supporting information

S1 Fig RHOF promotes actin reorganization and single cell migration of esophageal epithelial cells. (**A**) Compared to control cells, esophageal epithelial cells that expressed constitutively active *RhoF* (green) had reorganization of F-actin to the cell surface with small actin-rich surface projections as indicated by staining for phalloidin (red). DAPI staining is in blue. (**B**) Expression of constitutively active *RhoF* also increased single-cell migration of esophageal epithelial cells, as assessed by time-lapse microscopy.
(EPS)

S1 File Completed ARRIVE Guidelines Checklist. (PDF)

## References

[1] Peery AF, Dellon ES, Lund J, Crockett SD, McGowan CE, Bulsiewicz WJ, et al Burden of gastrointestinal disease in the United States: 2012 update. Gastroenterology. 2012;143(5):1179–87 e1-3. Epub 2012/08/14. 10.1053/j.gastro.2012.08.002 22885331 PMC3480553

[2] Abnet CC, Arnold M, Wei W-Q. Epidemiology of Esophageal Squamous Cell Carcinoma. Gastroenterology. 10.1053/j.gastro.2017.08.023 28823862 PMC5836473

[3] Torre LA, Bray F, Siegel RL, Ferlay J, Lortet-Tieulent J, Jemal A. Global cancer statistics, 2012. CA Cancer J Clin. 2015;65(2):87–108. Epub 2015/02/06. 10.3322/caac.21262 . 25651787

[4] Enzinger PC, Mayer RJ. Esophageal cancer. N Engl J Med. 2003;349(23):2241–52. 10.1056/NEJMra035010 . 14657432

[5] Lin EW, Karakasheva TA, Hicks PD, Bass AJ, Rustgi AK. The tumor microenvironment in esophageal cancer. Oncogene. 2016;35(41):5337–49. 10.1038/onc.2016.34 26923327 PMC5003768

[6] Tetreault MP, Weinblatt D, Ciolino JD, Klein-Szanto AJ, Sackey BK, Twyman-Saint Victor C, et al Esophageal Expression of Active IkappaB Kinase-beta in Mice Up-Regulates Tumor Necrosis Factor and Granulocyte-Macrophage Colony-Stimulating Factor, Promoting Inflammation and Angiogenesis. Gastroenterology. 2016;150(7):1609–19 e11. Epub 2016/02/21. S0016-5085(16)00216-X [pii] 10.1053/j.gastro.2016.02.025 26896735 PMC4909513

[7] Tetreault MP, Wang ML, Yang Y, Travis J, Yu QC, Klein-Szanto AJ, et al Klf4 overexpression activates epithelial cytokines and inflammation-mediated esophageal squamous cell cancer in mice. Gastroenterology. 2010;139(6):2124–34. Epub 2010/09/08. S0016-5085(10)01298-9 [pii] 10.1053/j.gastro.2010.08.048 . 20816834 PMC3457785

[8] Stairs DB, Bayne LJ, Rhoades B, Vega ME, Waldron TJ, Kalabis J, et al Deletion of p120-catenin results in a tumor microenvironment with inflammation and cancer that establishes it as a tumor suppressor gene. Cancer Cell. 2011;19(4):470–83. Epub 2011/04/13. S1535-6108(11)00083-3 [pii] 10.1016/j.ccr.2011.02.007 21481789 PMC3077713

[9] Davis BP, Rothenberg ME. Mechanisms of Disease of Eosinophilic Esophagitis. Annual review of pathology. 2016;11:365–93. Epub 2016/03/02. 10.1146/annurev-pathol-012615-044241 26925500 PMC4918086

[10] Mishra A. Significance of Mouse Models in Dissecting the Mechanism of Human Eosinophilic Gastrointestinal Diseases (EGID). Journal of gastroenterology and hepatology research. 2013;2(11):845–53. 10.6051/j.issn2224-3992.2013.02.343 PMC4391819. 25866707 PMC4391819

[11] DiDonato JA, Mercurio F, Karin M. NF-kappaB and the link between inflammation and cancer. Immunological reviews. 2012;246(1):379–400. Epub 2012/03/23. 10.1111/j.1600-065X.2012.01099.x . 22435567

[12] Gambhir S, Vyas D, Hollis M, Aekka A, Vyas A. Nuclear factor kappa B role in inflammation associated gastrointestinal malignancies. World journal of gastroenterology. 2015;21(11):3174–83. Epub 2015/03/26. 10.3748/wjg.v21.i11.3174 25805923 PMC4363746

[13] Fang Y, Chen H, Hu Y, Djukic Z, Tevebaugh W, Shaheen NJ, et al Gastroesophageal reflux activates the NF-kappaB pathway and impairs esophageal barrier function in mice. American journal of physiology Gastrointestinal and liver physiology. 2013;305(1):G58–65. Epub 2013/05/04. 10.1152/ajpgi.00438.2012 23639809 PMC3725692

[14] Chandramouleeswaran PM, Shen D, Lee AJ, Benitez A, Dods K, Gambanga F, et al Preferential Secretion of Thymic Stromal Lymphopoietin (TSLP) by Terminally Differentiated Esophageal Epithelial Cells: Relevance to Eosinophilic Esophagitis (EoE). PloS one. 2016;11(3):e0150968 Epub 2016/03/19. 10.1371/journal.pone.0150968 26992000 PMC4798725

[15] Lin C, Song L, Gong H, Liu A, Lin X, Wu J, et al Nkx2-8 downregulation promotes angiogenesis and activates NF-kappaB in esophageal cancer. Cancer research. 2013;73(12):3638–48. Epub 2013/04/23. 10.1158/0008-5472.CAN-12-4028 . 23604637

[16] Gong H, Song L, Lin C, Liu A, Lin X, Wu J, et al Downregulation of miR-138 Sustains NF-κB Activation and Promotes Lipid Raft Formation in Esophageal Squamous Cell Carcinoma. Clinical Cancer Research. 2013;19(5):1083–93. 10.1158/1078-0432.CCR-12-3169 23319823

[17] Persad R, Huynh HQ, Hao L, Ha JR, Sergi C, Srivastava R, et al Angiogenic remodeling in pediatric EoE is associated with increased levels of VEGF-A, angiogenin, IL-8, and activation of the TNF-alpha-NFkappaB pathway. Journal of pediatric gastroenterology and nutrition. 2012;55(3):251–60. Epub 2012/02/15. 10.1097/MPG.0b013e31824b6391 . 22331014

[18] Lim DM, Narasimhan S, Michaylira CZ, Wang M-L. TLR3-mediated NF-κB signaling in human esophageal epithelial cells. American Journal of Physiology—Gastrointestinal and Liver Physiology. 2009;297(6):G1172–G80. 10.1152/ajpgi.00065.2009 19779021 PMC2850089

[19] Rafiee P, Nelson VM, Manley S, Wellner M, Floer M, Binion DG, et al Effect of curcumin on acidic pH-induced expression of IL-6 and IL-8 in human esophageal epithelial cells (HET-1A): role of PKC, MAPKs, and NF-kappaB. American journal of physiology Gastrointestinal and liver physiology. 2009;296(2):G388–98. Epub 2008/12/17. 10.1152/ajpgi.90428.2008 . 19074641

[20] Kang MR, Kim MS, Kim SS, Ahn CH, Yoo NJ, Lee SH. NF-kappaB signalling proteins p50/p105, p52/p100, RelA, and IKKepsilon are over-expressed in oesophageal squamous cell carcinomas. Pathology. 2009;41(7):622–5. Epub 2009/12/17. 10.3109/00313020903257756 . 20001340

[21] Ghaleb AM, Yang VW. Kruppel-like factor 4 (KLF4): What we currently know. Gene. 2017;611:27–37. Epub 2017/02/27. 10.1016/j.gene.2017.02.025 28237823 PMC5391259

[22] Kapoor N, Niu J, Saad Y, Kumar S, Sirakova T, Becerra E, et al Transcription factors STAT6 and KLF4 implement macrophage polarization via the dual catalytic powers of MCPIP. Journal of immunology (Baltimore, Md: 1950). 2015;194(12):6011–23. Epub 2015/05/03. 10.4049/jimmunol.1402797 25934862 PMC4458412

[23] Liao X, Sharma N, Kapadia F, Zhou G, Lu Y, Hong H, et al Kruppel-like factor 4 regulates macrophage polarization. J Clin Invest. 2011;121(7):2736–49. Epub 2011/06/15. 10.1172/JCI45444 21670502 PMC3223832

[24] Feinberg MW, Cao Z, Wara AK, Lebedeva MA, Senbanerjee S, Jain MK. *Krüppel*-like factor 4 is a mediator of proinflammatory signaling in macrophages. The Journal of biological chemistry. 2005;280(46):38247–58. 10.1074/jbc.M509378200 . 16169848

[25] Kurotaki D, Osato N, Nishiyama A, Yamamoto M, Ban T, Sato H, et al Essential role of the IRF8-KLF4 transcription factor cascade in murine monocyte differentiation. Blood. 2013;121(10):1839–49. Epub 2013/01/16. 10.1182/blood-2012-06-437863 23319570 PMC3591803

[26] Alder JK, Georgantas RW 3rd, Hildreth RL Kaplan IM, Morisot S, Yu X, et al *Krüppel*-like factor 4 is essential for inflammatory monocyte differentiation in vivo. Journal of immunology (Baltimore, Md: 1950). 2008;180(8):5645–52. . 18390749 10.4049/jimmunol.180.8.5645 PMC3074963

[27] Rosenzweig JM, Glenn JD, Calabresi PA, Whartenby KA. KLF4 modulates expression of IL-6 in dendritic cells via both promoter activation and epigenetic modification. The Journal of biological chemistry. 2013;288(33):23868–74. Epub 2013/07/13. 10.1074/jbc.M113.479576 23846700 PMC3745333

[28] Hartmann P, Zhou Z, Natarelli L, Wei Y, Nazari-Jahantigh M, Zhu M, et al Endothelial Dicer promotes atherosclerosis and vascular inflammation by miRNA-103-mediated suppression of KLF4. Nature communications. 2016;7:10521 Epub 2016/02/04. 10.1038/ncomms10521 26837267 PMC4742841

[29] Shen B, Smith RS Jr., Hsu YT, Chao L, Chao J. Kruppel-like factor 4 is a novel mediator of Kallistatin in inhibiting endothelial inflammation via increased endothelial nitric-oxide synthase expression. The Journal of biological chemistry. 2009;284(51):35471–8. Epub 2009/10/28. 10.1074/jbc.M109.046813 19858207 PMC2790976

[30] Kaushik DK, Thounaojam MC, Kumawat KL, Gupta M, Basu A. Interleukin-1beta orchestrates underlying inflammatory responses in microglia via Kruppel-like factor 4. J Neurochem. 2013;127(2):233–44. Epub 2013/07/31. 10.1111/jnc.12382 . 23895397

[31] Ghaleb AM, Laroui H, Merlin D, Yang VW. Genetic deletion of Klf4 in the mouse intestinal epithelium ameliorates dextran sodium sulfate-induced colitis by modulating the NF-kappaB pathway inflammatory response. Inflamm Bowel Dis. 2014;20(5):811–20. Epub 2014/04/01. 10.1097/MIB.0000000000000022 24681655 PMC4091934

[32] Sun S-C. The non-canonical NF-κB pathway in immunity and inflammation. Nature Reviews Immunology. 2017;17:545 10.1038/nri.2017.52 28580957 PMC5753586

[33] Tetreault MP, Yang Y, Travis J, Yu QC, Klein-Szanto A, Tobias JW, et al Esophageal squamous cell dysplasia and delayed differentiation with deletion of *Krüppel*-like factor 4 in murine esophagus. Gastroenterology. 2010;139(1):171–81. Epub 2010/03/30. S0016-5085(10)00481-6 [pii] 10.1053/j.gastro.2010.03.048 . 20347813 PMC3265336

[34] Perona R, Montaner S, Saniger L, Sanchez-Perez I, Bravo R, Lacal JC. Activation of the nuclear factor-kappaB by Rho, CDC42, and Rac-1 proteins. Genes Dev. 1997;11(4):463–75. Epub 1997/02/15. . 9042860 10.1101/gad.11.4.463

[35] Tong L, Tergaonkar V. Rho protein GTPases and their interactions with NFkappaB: crossroads of inflammation and matrix biology. Bioscience reports. 2014;34(3). Epub 2014/06/01. 10.1042/bsr20140021 24877606 PMC4069681

[36] Perez-Moreno M, Davis MA, Wong E, Pasolli HA, Reynolds AB, Fuchs E. p120-catenin mediates inflammatory responses in the skin. Cell. 2006;124(3):631–44. Epub 2006/02/14. S0092-8674(06)00008-0 [pii] 10.1016/j.cell.2005.11.043 16469707 PMC2443688

[37] Haga RB, Ridley AJ. Rho GTPases: Regulation and roles in cancer cell biology. Small GTPases. 2016;7(4):207–21. 10.1080/21541248.2016.1232583 . 27628050 PMC5129894

[38] Lehman HL, Kidacki M, Warrick JI, Stairs DB. NFkB hyperactivation causes invasion of esophageal squamous cell carcinoma with EGFR overexpression and p120-catenin down-regulation. Oncotarget. 2018;9(13):11180–96. Epub 2018/03/16. 10.18632/oncotarget.24358 29541406 PMC5834278

[39] Aspenstrom P. Atypical Rho GTPases RhoD and Rif integrate cytoskeletal dynamics and membrane trafficking. Biol Chem. 2014;395(5):477–84. Epub 2014/03/14. 10.1515/hsz-2013-0296 /j/bchm.just-accepted/hsz-2013-0296/hsz-2013-0296.xml [pii]. . 24622787

[40] Ellis S, Mellor H. The novel Rho-family GTPase rif regulates coordinated actin-based membrane rearrangements. Curr Biol. 2000;10(21):1387–90. Epub 2000/11/21. S0960-9822(00)00777-6 [pii]. . 11084341 10.1016/s0960-9822(00)00777-6

[41] Fan L, Mellor H. The small Rho GTPase Rif and actin cytoskeletal remodelling. Biochem Soc Trans. 2012;40(1):268–72. Epub 2012/01/21. BST20110625 [pii] 10.1042/BST20110625 . 22260703

[42] Fan L, Pellegrin S, Scott A, Mellor H. The small GTPase Rif is an alternative trigger for the formation of actin stress fibers in epithelial cells. J Cell Sci. 2010;123(Pt 8):1247–52. Epub 2010/03/18. jcs.061754 [pii] 10.1242/jcs.061754 20233848 PMC2848113

[43] Pellegrin S, Mellor H. The Rho family GTPase Rif induces filopodia through mDia2. Curr Biol. 2005;15(2):129–33. Epub 2005/01/26. S096098220500031X [pii] 10.1016/j.cub.2005.01.011 . 15668168

[44] Hodge RG, Ridley AJ. Regulating Rho GTPases and their regulators. Nature reviews Molecular cell biology. 2016;17(8):496–510. Epub 2016/06/16. 10.1038/nrm.2016.67 . 27301673

[45] Hall A. Rho family GTPases. Biochemical Society Transactions. 2012;40(6):1378 10.1042/BST20120103 23176484

[46] Goggs R, Savage JS, Mellor H, Poole AW. The small GTPase Rif is dispensable for platelet filopodia generation in mice. PloS one. 2013;8(1):e54663 Epub 2013/01/30. 10.1371/journal.pone.0054663 23359340 PMC3554654

[47] Liacouras CA, Furuta GT, Hirano I, Atkins D, Attwood SE, Bonis PA, et al Eosinophilic esophagitis: updated consensus recommendations for children and adults. The Journal of allergy and clinical immunology. 2011;128(1):3–20.e6; quiz 1–2. Epub 2011/04/12. 10.1016/j.jaci.2011.02.040 . 21477849

[48] Yang Y, Nakagawa H, Tetreault MP, Billig J, Victor N, Goyal A, et al Loss of transcription factor KLF5 in the context of p53 ablation drives invasive progression of human squamous cell cancer. Cancer research. 2011;71(20):6475–84. Epub 2011/08/27. 10.1158/0008-5472.CAN-11-1702 21868761 PMC3193554

[49] Yang Y, Goldstein BG, Chao H-H, Katz J. KLF4 and KLF5 regulate proliferation, Apoptosis and invasion in esophageal cancer cells. Cancer Biology & Therapy. 2005;4(11):1216–21. 10.4161/cbt.4.11.2090 16357509

[50] Yang Y, Goldstein BG, Nakagawa H, Katz JP. *Krüppel*-like factor 5 activates MEK/ERK signaling via EGFR in primary squamous epithelial cells. Faseb J. 2007;21(2):543–50. 10.1096/fj.06-6694com . 17158781

[51] Harada H, Nakagawa H, Oyama K, Takaoka M, Andl CD, Jacobmeier B, et al Telomerase induces immortalization of human esophageal keratinocytes without p16INK4a inactivation. Mol Cancer Res. 2003;1(10):729–38. Epub 2003/08/27. . 12939398

[52] Ren XD, Schwartz MA. Determination of GTP loading on Rho. Methods in enzymology. 2000;325:264–72. Epub 2000/10/19. . 11036609 10.1016/s0076-6879(00)25448-7

[53] Kalabis J, Wong GS, Vega ME, Natsuizaka M, Robertson ES, Herlyn M, et al Isolation and characterization of mouse and human esophageal epithelial cells in 3D organotypic culture. Nat Protoc. 2012;7(2):235–46. Epub 2012/01/14. 10.1038/nprot.2011.437 22240585 PMC3505594

[54] Laczkó D, Wang F, Johnson FB, Jhala N, Rosztóczy A, Ginsberg GG, et al Modeling Esophagitis Using Human Three-Dimensional Organotypic Culture System. The American Journal of Pathology. 2017;187(8):1787–99. 10.1016/j.ajpath.2017.04.013 28627413 PMC5974532

[55] Shaverdashvili K, Wong P, Ma J, Zhang K, Osman I, Bedogni B. MT1-MMP modulates melanoma cell dissemination and metastasis through activation of MMP2 and RAC1. Pigment cell & melanoma research. 2014;27(2):287–96. Epub 2014/01/07. 10.1111/pcmr.12201 . 24387669

[56] Shaverdashvili K, Zhang K, Osman I, Honda K, Jobava R, Bedogni B. MT1-MMP dependent repression of the tumor suppressor SPRY4 contributes to MT1-MMP driven melanoma cell motility. Oncotarget. 2015;6(32):33512–22. Epub 2015/09/24. 10.18632/oncotarget.5258 26392417 PMC4741782

[57] Chon JH, Vizena AD, Rock BM, Chaikof EL. Characterization of single-cell migration using a computer-aided fluorescence time-lapse videomicroscopy system. Analytical biochemistry. 1997;252(2):246–54. Epub 1997/11/05. 10.1006/abio.1997.2321 . 9344410

[58] Lutz S, Mohl M, Rauch J, Weber P, Wieland T. RhoGEF17, a Rho-specific guanine nucleotide exchange factor activated by phosphorylation via cyclic GMP-dependent kinase Ialpha. Cell Signal. 2013;25(3):630–8. Epub 2012/12/01. S0898-6568(12)00319-1 [pii] 10.1016/j.cellsig.2012.11.016 . 23195829

[59] Goggs R, Williams Christopher M, Mellor H, Poole Alastair W. Platelet Rho GTPases–a focus on novel players, roles and relationships. Biochemical Journal. 2015;466(Pt 3):431–42. 10.1042/BJ20141404 PMC4357237. 25748676 PMC4357237

[60] Warner H, Wilson BJ, Caswell PT. Control of adhesion and protrusion in cell migration by Rho GTPases. Current opinion in cell biology. 2018;56:64–70. Epub 2018/10/07. 10.1016/j.ceb.2018.09.003 . 30292078 PMC6368645

[61] Cheng E, Souza RF, Spechler SJ. Tissue remodeling in eosinophilic esophagitis. American journal of physiology Gastrointestinal and liver physiology. 2012;303(11):G1175–87. Epub 2012/09/29. 10.1152/ajpgi.00313.2012 23019192 PMC3532456

[62] Jiang M, Ku WY, Zhou Z, Dellon ES, Falk GW, Nakagawa H, et al BMP-driven NRF2 activation in esophageal basal cell differentiation and eosinophilic esophagitis. J Clin Invest. 2015;125(4):1557–68. Epub 2015/03/17. 10.1172/JCI78850 25774506 PMC4396468

[63] Hanahan D, Weinberg RA. Hallmarks of cancer: the next generation. Cell. 2011;144(5):646–74. Epub 2011/03/08. 10.1016/j.cell.2011.02.013 . 21376230

[64] Aceves SS. Remodeling and fibrosis in chronic eosinophil inflammation. Digestive diseases (Basel, Switzerland). 2014;32(1–2):15–21. Epub 2014/03/08. 10.1159/000357004 24603375 PMC4037288

[65] Liu K, Jiang M, Lu Y, Chen H, Sun J, Wu S, et al Sox2 cooperates with inflammation-mediated Stat3 activation in the malignant transformation of foregut basal progenitor cells. Cell Stem Cell. 2013;12(3):304–15. Epub 2013/03/12. 10.1016/j.stem.2013.01.007 23472872 PMC3594795

[66] Perez-Moreno M, Song W, Pasolli HA, Williams SE, Fuchs E. Loss of p120 catenin and links to mitotic alterations, inflammation, and skin cancer. Proc Natl Acad Sci U S A. 2008;105(40):15399–404. Epub 2008/09/24. 0807301105 [pii] 10.1073/pnas.0807301105 18809907 PMC2547465

[67] Biro M, Munoz MA, Weninger W. Targeting Rho-GTPases in immune cell migration and inflammation. British journal of pharmacology. 2014;171(24):5491–506. Epub 2014/02/28. 10.1111/bph.12658 24571448 PMC4282076

[68] Pajic M, Herrmann D, Vennin C, Conway JR, Chin VT, Johnsson AK, et al The dynamics of Rho GTPase signaling and implications for targeting cancer and the tumor microenvironment. Small GTPases. 2015;6(2):123–33. Epub 2015/06/24. 10.4161/21541248.2014.973749 26103062 PMC4601362

